# Selection-Driven Gene Loss in Bacteria

**DOI:** 10.1371/journal.pgen.1002787

**Published:** 2012-06-28

**Authors:** Sanna Koskiniemi, Song Sun, Otto G. Berg, Dan I. Andersson

**Affiliations:** 1 Department of Medical Biochemistry and Microbiology, Uppsala University, Uppsala, Sweden; 2 Department of Cell and Molecular Biology, Uppsala University, Uppsala, Sweden; Universidad de Sevilla, Spain

## Abstract

Gene loss by deletion is a common evolutionary process in bacteria, as exemplified by bacteria with small genomes that have evolved from bacteria with larger genomes by reductive processes. The driving force(s) for genome reduction remains unclear, and here we examined the hypothesis that gene loss is selected because carriage of superfluous genes confers a fitness cost to the bacterium. In the bacterium *Salmonella enterica*, we measured deletion rates at 11 chromosomal positions and the fitness effects of several spontaneous deletions. Deletion rates varied over 200-fold between different regions with the replication terminus region showing the highest rates. Approximately 25% of the examined deletions caused an increase in fitness under one or several growth conditions, and after serial passage of wild-type bacteria in rich medium for 1,000 generations we observed fixation of deletions that substantially increased bacterial fitness when reconstructed in a non-evolved bacterium. These results suggest that selection could be a significant driver of gene loss and reductive genome evolution.

## Author Summary

The mechanisms and driving forces involved in reductive evolution and loss of non-used gene functions are still largely unknown. Since most deletions are thought to have a negative effect on fitness, it is thought that deletions accumulate by chance, via non-adaptive genetic drift combined with an associated underlying mutational deletion bias. Another possibility is that an adaptive process drives gene loss because superfluous genes confer a fitness cost to the bacterium, providing positive selection for gene loss. Here we have tested the latter idea, and our results show that a surprisingly high fraction of random deletions introduced into the Salmonella chromosome do in fact increase fitness as measured by exponential growth rate. Furthermore, when Salmonella is grown for many generations in a rich growth medium, fitness-increasing deletions accumulate in the population. Our results suggest that selection as well as non-adaptive processes might drive genome reduction and that for certain chromosomal regions gene loss may occur by a rapid adaptive process.

## Introduction

A central biological question is how genomes evolve with respect to size and gene content and which factors affect and constrain this evolution. Clearly many bacterial genomes are in continuous flux with respect to genome size and a number of processes, including gene loss, gene duplications, gene fusions, de novo development of new functions and horizontal gene transfer, will affect gene content. With regard to gene loss it remains unclear which are the major factors that drive this process. The pioneering studies of Zamenhof and Eichhorn [1], Dykhuizen [2] and Koch [3] showed that reduced expression of certain biosynthetic and catabolic operons could for unknown reasons result in an increased fitness. Similarly, in *E. coli* evolved for 40,000 generations in the laboratory several deletions were identified [4] and a few of these were later confirmed to confer a beneficial effect [5]. Furthermore, it has been shown that inactivation/loss of the *cadA* gene, encoding lysine decarboxylase, can increase virulence of Shigella, providing a potential driving force for gene loss [6]–[8]. For bacterial obligate endosymbionts and intracellular pathogens that have evolved from free-living bacterial species with large genomes, their small genomes are most likely the result of increased genetic drift associated with an intracellular lifestyle and population bottlenecks [9]–[18]. Combined with relaxed selection for many bacterial functions in the intracellular environment [11]–[16], an underlying mutational deletion bias [12]–[14], [18]–[20] and restricted rates of horizontal gene transfer inside host cells [15], [16], [21], deleterious and neutral deletions will accumulate over time in a ratchet-like manner [9]–[11], [14] and result in smaller genomes. The biased mutation spectrum and increased mutation rate possibly result from the deletion of DNA repair genes, a gene class that is reduced or absent in many bacteria with reduced genomes [16], [19], [22]–[26]. We showed in previous experiments that under in vitro conditions that mimic the intracellular environment (i.e. population bottlenecks, relaxed selection for certain bacterial genes and absence of HGT), extensive gene loss by large deletions can occur over short time scales [27]. However, these findings do not exclude the possibility that adaptive processes contribute to gene deletion as well, and it has been suggested that for certain free-living bacteria selection might drive genome reduction [28]–[30]. To examine the importance of selection as a driver of gene loss, we determined how frequently and to what extent gene loss could confer an increase in fitness under controlled experimental conditions.

## Results/Discussion

### Genome-wide deletion rates

**Figure 1:**
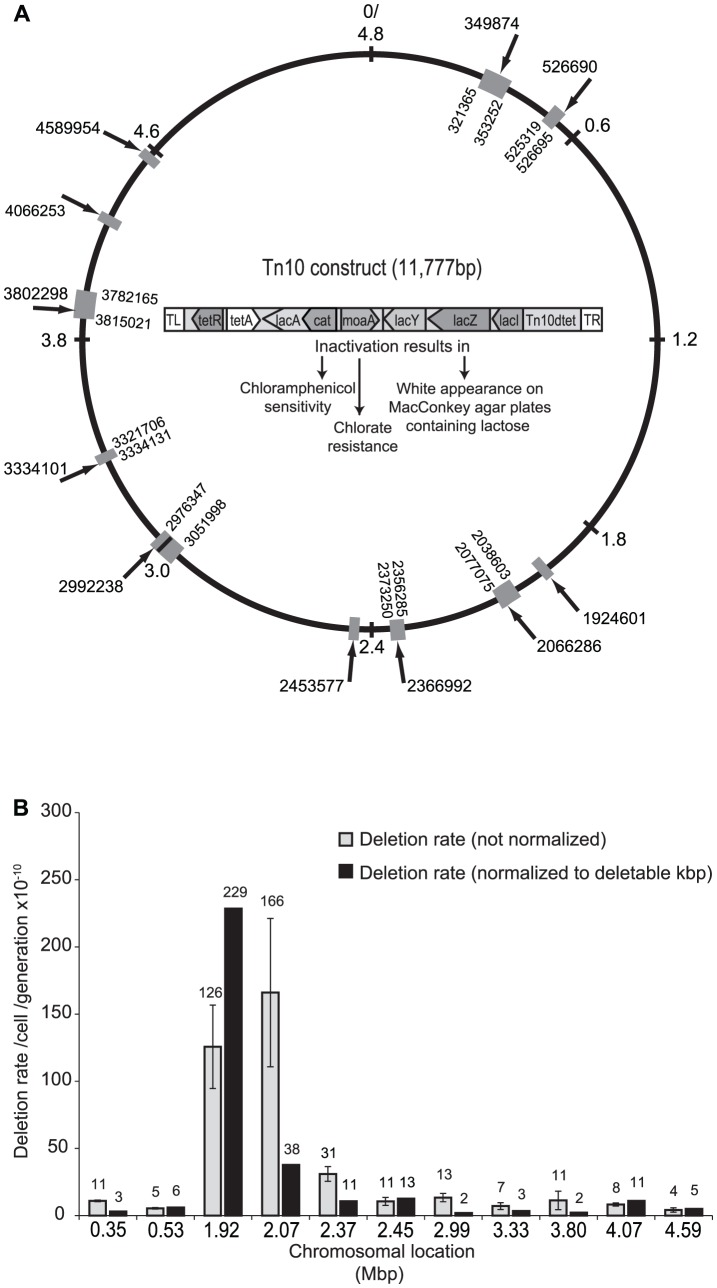
Chromosomal locations of the Tn*10* construct and deletion rates. (*A*) Schematic representation of the *Salmonella typhimurium* LT chromosome and the structure and insertion points of the Tn*10* construct used for measurements of deletion rates. Numbers and arrows outside the chromosome ring indicate the Tn*10* insertion point. The relative size of the grey box and the numbers on the inside of the ring indicate the size of the deletions isolated in that specific region. When numbers are absent this indicate that the deletions were internal to the Tn*10* construct. In the middle, the structure and gene content of the Tn*10* construct and the resulting phenotypes of loss of the *cat, moaA* and *lacZ* genes, respectively, are shown. (*B*) Deletion rates at 11 different chromosomal regions. Standard errors are indicated. The normalization procedure is described in Text S1_ENREF_20. Compare with Figure S4 for a different normalization method (Text S1).

The rate of selection-driven gene loss will be determined by two main parameters: the deletion rate at different chromosomal regions and the resulting fitness effects of these deletions. To allow determination of deletion rates at any location and to isolate spontaneous deletions, we constructed a Tn*10* transposon derivative that is defective for transposition and that carries the *lacZYA* operon with a *moaA* (encodes an enzyme involved in molybdate cofactor biosynthesis) and a chloramphenicol resistance gene (*cat*) inserted into the *lacA* gene (Figure 1A and Text S1_ENREF_20). The engineered transposon was allowed to transpose into the chromosome of a strain where the chromosomal copy of the native *moaA* gene had been deleted, and 11 transposon insertions distributed over the Salmonella chromosome were chosen to measure the local deletion rates. These transposon insertions had no detectable effect on bacterial growth rates as compared to a wild type strain without the transposon. By selecting for loss of the *moaA* marker in the transposon (confers chlorate resistance). and simultaneously screening for the loss of the *lacZY* genes (white colonies on McConkey agar plates), spontaneous deletions were detected. To further confirm that the white, chlorate resistant colonies represented true deletions, loss of the *cat* gene was determined by lack of growth on medium supplemented with chloramphenicol. To be detected in this assay, a deletion has to remove at least 2 kbp of DNA (i.e. *lacY, moaA* and *cat*) and the upper limit deletion size will be determined by how much non-essential DNA flanks the insertion point of the transposon. The apparent deletion rate at each of 11 examined chromosomal locations varied from 0.5×10^−9^ to 2.2×10^−8^/cell/generation (Figure 1B). However, as deletion rates are expected to be higher when the transposon is inserted in a region containing more non-essential deletable DNA, we corrected for this effect by normalizing the deletion rate at each location to the experimentally identified deletable region, i.e. the largest deletion found at each specific region (Materials and Methods)_ENREF_20. After this normalization, deletion rates were found to vary between 0.5×10^−11^ and 1.25×10^−9^/cell/generation/deletable kbp of DNA, resulting in a 225-fold difference in deletion rates when comparing different regions (Figure 1B). Using a more realistic normalization procedure, similar results were obtained (Text S1 and Figure S1) The three insertions with the highest deletion rates were all located in the 2 Mbp region of the Salmonella chromosome, suggesting a potential hotspot for deletion formation near the replication terminus region, whereas the rates were lower and similar around the remainder of the chromosome. The isolated deletions ranged in size from 2–67 kbp and whole-genome sequencing (Materials and Methods) _ENREF_20of 30 unique deletion mutants showed that short homologies (6–15 bp) were present at the endpoints for the majority (19/30) of the analyzed deletions (Table S1 and Figure S2). For the remaining deletions (11/30), ≤4 bp or no homology was found at the endpoints. As RecA requires at least 25 bp to mediate its action, these findings imply that spontaneous deletion formation occur mainly via RecA-independent homologous recombination [31], [32].

### Fitness effects of deletions obtained from 11 chromosomal positions

**Figure 2:**
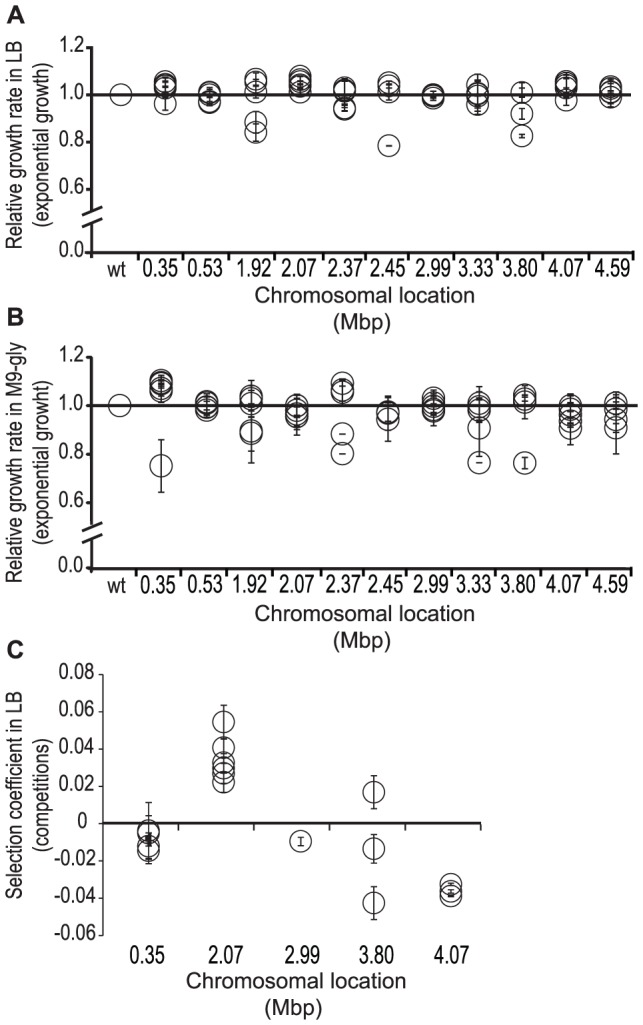
Relative fitness of mutants with deletion of different chromosomal regions. (*A*) Relative exponential growth rates (parental strain set to 1.0) in single cultures for mutants with deletion of different chromosomal regions during growth in rich LB medium. (*B*) Relative exponential growth rates (parental strain set to 1.0) in single cultures for mutants with deletion of different chromosomal regions during growth in minimal (M9) glycerol medium. (*C*) Selection coefficients of deletion mutants obtained from competition experiments in rich LB medium with an isogenic parental control (defined as s = 0).

Apart from the deletions identified at the transposon insertion point, these 30 mutant strains had no other sequence changes, allowing us to determine how deletions that were isolated at the different chromosomal locations affected bacterial fitness. Fitness was initially measured as growth rate during exponential growth in rich LB media and M9-media supplemented with glycerol for 5 different deletions from each of the 11 different chromosomal regions, representing a total of 55 mutants (Materials and Methods)_ENREF_20. Relative growth rates (wild type set to 1.0) varied between 0.8 and 1.1 in LB and 0.7 to 1.1 in M9-glycerol for the different mutants. No correlation between deletion size and fitness effects could be detected but instead the fitness effects of the different deletions grouped together for each specific region (Figure 2A–2B and Table S1). For 15/55 deletion mutants (varying in size from 18 to 44 kbp and located at 0.35, 2.07 Mbp and 3.80 Mbp) an increased growth rates were observed in either rich or poor growth media, and for 3/55 mutants growth rates were increased in both media. To increase assay sensitivity, we performed competition experiments in which a composite fitness during the entire growth curve was measured. These competitions allowed the detection of fitness differences as small as s = 0.003 [33]. A subset of 13 deletion mutants was competed against the parental strain for 50 generations in LB (Materials and Methods)_ENREF_20. Of these 13 mutants, eight showed a significantly increased exponential growth rate in single culture and five were indistinguishable from the parent strain. Six out of thirteen deletion mutants, representing deletions from two different chromosomal positions (2.07 Mbp and 3.80 Mbp), showed an increased competitive fitness (Table S1 and Figure 2C). The remaining seven deletion mutants showed no increase in competitive ability, even though they had a faster exponential growth rate in single cultures in LB- or M9-medium or both. By plotting the change in selection coefficient (Δs) for each mutant grown in single cultures in LB- versus M9-medium supplemented with glycerol and for each mutant grown in single culture versus competitions in LB-medium (Figure S3A–S3B) it can be seen that the fitness-increasing effect of these different deletions is conditional and depends on both growth media and assay condition. These findings also provide an explanation for why bacteria maintain genes that have, at least in specific laboratory environments, fitness costs. Thus, it is likely that the costly genes are beneficial in certain natural environments and therefore selectively maintained.

### Selection for fitness-increasing deletions during repeated serial passage

Since 13/55 deletions isolated from three different chromosomal positions increased the fitness of the cells under at least one of three tested conditions, this indicated that fitness-increasing deletions are common. A prediction from this finding is that continuous growth of wild type bacteria would result in selection and fixation of mutant strains carrying deletions. Six independent lineages of wild type bacteria were grown by repeated serial passage for 1000 generations in rich LB medium (Materials and Methods)_ENREF_20. Competition experiments showed that all evolved lineages could outcompete the parental strain, indicating that they had acquired mutations that increased fitness. As shown by whole genome sequencing, the genomes of all six evolved lineages contained more than one type of mutation, including single nucleotide polymorphisms and deletions (Table S2), and any of these mutations alone or in combination could potentially contribute to the increased competitive ability of the evolved strains. To specifically determine if any of the deletions contributed to the faster growth, we reconstructed six different deletion mutations in the non-evolved parental strain by lambda red recombineering (Materials and Methods)_ENREF_20. As shown by competition experiments in rich LB medium, two out of six of these deletion mutations increased fitness with 4.7% and 3.2%, respectively. One deletion removed 5.2 kbp of DNA, including the *uvrC, uvrY, yecF, sdiA, yecC, yecS* genes, and the second removed 54 bp in the *fliG* gene. The remaining four deletions had either no effect on fitness or reduced fitness when introduced into a wild type genetic background. Since the probability of fixation of a neutral or deleterious deletion during 1000 generations of serial passage is essentially nil (Text S1), it is likely that the singly neutral/deleterious deletions were also selected in this experiment but their ability to increase fitness is only observed in combination with one or several of the other mutations present in these serially passaged strains.

### Modeling of fixation rates of deletions

These results indicate that selection could be a significant driver of gene loss and with the observed deletion rates (0.5×10^−11^ to 1.25×10^−9^/cell/generation/deletable kbp of DNA) and substantial fitness increases (Δs-values between +0.017 and +0.054 as determined by single cultures and competition experiments), it is expected that gene loss can occur very rapidly also in natural populations. Modeling the rate of fixation for the parameter values determined in this study (Text S1), showed that take-over by deletion mutants could occur in less than 1,000 generations (Figure S4). From these results it is difficult to assess the relative contributions of drift and selection to gene loss in natural settings but it is likely that both processes contribute with different rates and dependence on population structure. Thus, with larger population sizes selection-driven gene loss is a faster process whereas with population bottlenecks genetic drift increases in importance. A key question is which fraction of all potential deletions increases fitness and how many are neutral or deleterious? Our results show that for large deletions (>2 kbp), fitness can be increased by deletions at 3 out of 11 random positions on the chromosome, whereas for eight regions deletions appear neutral or deleterious. This implies that for certain chromosomal regions gene loss may occur by a rapid adaptive process whereas for other regions a slower genetic drift process probably dominates.

### Why do these deletions affect fitness?

Since most of the deletions remove several genes (many of which have no assigned function) it is difficult to explain why a specific deletion will have a beneficial, neutral or deleterious effect on fitness. With regard to the beneficial deletions, one potential explanation is that loss of these genes result in decreased energy/mass expenditure on DNA, RNA and protein and a resulting faster growth rate because more resources can be allocated to other rate-limiting processes. This idea has previously been explored by in several studies where the effects on growth rate by specific mutations in anabolic/catabolic operons were examined [1]–[3]. As the cost of protein synthesis exceeds that of DNA and RNA for a given gene, expression of a non-used protein of length *L* amino acids by *n* copies – assuming that the protein has no other adverse effects – will reduce fitness by *s* = −*nL/r*_0_ where r_0_ = 10^9^ is the total number of amino acids incorporated into protein in a bacterial cell [34], [35]. Using published data on protein levels from *S. enterica* growing in LB medium [36], we calculated the *nL* values for the genes that were included in the different deletions (Table S3). Under the assumption that the biosynthetic resources spent on these non-used genes can be allocated to other rate-limiting processes (e.g. protein synthesis), for all deletions the observed beneficial effect on the growth rate is higher than expected, indicating that the growth-rate increasing effect is not only a consequence of reduced energy/mass expenditure on protein production but includes other effects as well [1]–[3]. For example, running the flagellum represents a substantial cost in terms of dissipated proton motive force [37] and it is possible that for the non-motile *fliG* deletion mutant, fitness is increased because PMF energy not spent on the flagella might instead be used to make ATP. It is notable that the calculated expected cost of running flagella is 4.5% of the cells total energy expenditure (Text S1), which corresponds well with the 3.2% fitness increase observed in the non-motile *fliG* deletion mutant. It is likely that the deletions in the 2.07 Mbp region (Figure 2A–2C) confer their fitness-increasing effect for the same reason since they remove all or parts of the *fli* operon and inactivate flagellum function.

### A deletion hotspot

Finally, from our genome-wide determination of deletion rates and previous studies [38], [39] it appears as if the terminus region is a hotspot for recombination, implying that genes in the replication terminus region are more prone to loss. For example, genome comparisons of natural populations of *Bartonella henselae* strains show that the terminus region is highly unstable with numerous deletions and inversions occurring over short evolutionary time scales, lending support to this notion [40].

## Materials and Methods

### Strains and media

The bacterial strains used in this study were derived from *Salmonella enterica* var *typhimurium* LT2 (designated *S. typhimurium* throughout this paper) and are listed in Table S4. Bacteria were grown in standard Luria Bertani-broth (LB) or M9 minimal media supplemented with 0.2% glucose or glycerol [41]. When grown overnight, bacteria were incubated in 37°C and liquid cultures were shaken at 200 rpm. Antibiotics concentrations used were as follows: ampicillin (Amp) 100 mg/L, chloramphenicol (Cam) 20 mg/L, tetracycline (Tet) 30 mg/L, in both agar plates and culture media.

### PCR, transductions, linear transformation, and strain constructions

All plasmids were prepared using the E.Z.N.A plasmid mini kit (Omega Bio-Tek) and transferred between strains using transformation or transduction. Deletion of the *moaA* gene in *S. typhimurium* were constructed by linear transformation as described previously in strain DA6196 carrying the lambda *red* system [42]. Linear DNA was produced by PCR using primers F: 5′-tgtaggctggagctgcttc-3′and R: 5′-catatgaatatcctcctta-3′ for Cam and Kan-casettes, with 40 bp of homologous DNA to insertion site flanking at the 5′ ends (Table S5). All primers were purchased from MWG-Biotech. As templates, plasmids pKD3 (Cam) and pKD4 (kan) were used to create the antibiotic resistance marker. All PCRs were run with Taq gold enzyme (Applied biosystems) according to the following protocol in a Geneamp 9700 (Applied biosystems); 94°C 2 min, then 31 rounds of 94°C 30 s, annealing (55–65°C) 30 s, elongation 72°C (30 s-2 min) and a final elongation at 72°C for 7 min before cooling down to 4°C. After PCR DNA was run on gel electrophoresis and reactions of the appropriate size were purified with a PCR purification kit (Fermentas). Resistance markers from pKD3 and pKD4 inserted by linear transformation included FRT-recombination sites present on the template plasmids. Resistance markers were removed from strains using plasmid pCP20 carrying the FLP-recombinase under thermal induction control [42].

### Deletion assay

The deletometer consists of one counter-selected marker and two selected markers that can be used to determine rates of spontaneous deletion formation. Loss-of-function mutations (e.g. deletions) in the *moaA* genes renders cells chlorate resistance due to inactivation of the molybdate biosynthesis pathway [43]. Any inactivating mutation in the *lac* operon will make the cells appear white on MacConkey agar plates containing lactose due to the inability to ferment lactose. Any mutant chlorate resistant white colony is more likely to have been formed due to a deletion that simultaneously remove both *moaA* and *lac* rather than two independent inactivating mutations in each of the two genes. To further assure that chlorate resistant white colonies are indeed deletions, we inserted a chloramphenicol resistance marker adjacent to the *moaA* gene, which in a deletion should be lost causing the cells to become chloramphenicol susceptible (Figure 1A). To determine the rate of deletion formation, bacteria were grown in nine independent 1 ml overnight cultures inoculated with 10^6^ cells, in M9 minimal media supplemented with 0.2% glucose. 900 ml from each overnight culture was plated on MacConkey agar plates supplemented with 0.2% lactose and 0.2% sodium chlorate. Plates were incubated at 37°C for 24 h anaerobically and then for 6 h aerobically to select for chlorate resistant colonies (*moaA* mutations). These colonies were then scored for white appearance (*lac* mutations) and white colonies that were chloramphenicol susceptible were confirmed as deletions [44]. For each independent culture the number of chlorate-resistant, white, chloramphenicol susceptible colonies was divided by the total number of cells plated and deletion rates were calculated with either the median or P_0_ method [45], [46].

### Determination of approximate deletion size

The sizes of the deletions in the respective region were estimated using pulsed field gel electrophoresis (PFGE). For each region, 5 to 9 strains harboring a deletion were included for the PFGE. Cells were grown overnight and 100 ml of the overnight culture was mixed with 100 ml 1.5% low melting point agarose (Sigma) and lysozyme (1 mg/L final concentration) (Fluka). The mixture was transferred into small plastic wells and allowed to solidify on ice for 30 min. The agarose plugs were incubated for 2 h at 37°C in 400 ml EC-buffer supplemented with lysozyme to a final concentration of 1 mg/l. After incubation at 37°C the agarose plugs were incubated in ES buffer supplemented with proteinase K (1 g/L final concentration) at 56°C for 24 h. This step was repeated once. Finally agarose plugs were transferred to 0.5 M EDTA, pH 8.0 and stored at 4°C. Before samples were run on a gel, small pieces of the agarose plugs were cut and washed 2 times in 1×TE, once in 0.1×TE and once in 1×RE buffer (restriction enzyme buffer). Then the DNA was cut inside the agarose plug with xbaI (Fermentas) in 1×RE buffer for 2 h at 37°C. The plugs were washed once in 0.5XTBE before mounted onto the comb with 1% SeaKem LE agarose in 0.5XTBE and the gel was poured. The gel was run at 6 V/cm, 120°, switching from 6.8 sec to 63.8 sec for 23 h at 15°C. Gels were dyed in ethidium bromide and photographed in UV light. After PFGE, 5 strains harbouring different types of deletions were picked from each region. DNA was prepared from these strains using the Promega wizard genomic DNA purification kit according to the manufacturer (Promega). Primers were designed to map the deleted regions at 10 kb specificity. PCR was run as described earlier and for each set of primers wild type DNA (DA6192) was used as a positive control and dH_2_O as negative control for the PCR. Genes were determined to either be absent (deleted) or present, giving an approximated size of each individual deletion.

### Mapping of exact deletion endpoints by whole-genome sequencing

Genomic DNA from 25 independent strains carrying deletions at 10 different locations was prepared using the Qiagen genomic tip 500 G kit according to instruction from the manufacturer (Qiagen). Sequencing libraries were prepared from 5 µg of gDNA according to the manufacturer's guide Multiplexing sample preparation guide #1005361 revC using the NEBNext DNA sample prep reagents set 1 (New England BioLabs). Briefly, the DNA was fragmented using nebulization with compressed air at 32–35psi for 6 min. The DNA fragments were end-repaired using T4 DNA polymerase, Klenow DNA polymerase and T4 polynucleotide kinase (PNK), followed by purification on a QIAquick PCR purification column (Qiagen). An A-base was ligated to the blunt ends of the DNA fragments using the Klenow DNA polymerase and the sample was purified using a MinElute PCR purification column (Qiagen). Adapters for sequencing were ligated to the DNA fragments and the library was size selected on an agarose gel. A 200 bp fragment was excised from the gel, purified using a QIAGEN gel extraction column and amplified for 18 cycles of PCR, followed by purification using a Qiaquick or MinElute PCR Purification column. The quality of the library was evaluated using the Agilent Technologies 2100 Bioanalyzer and a DNA 1000-kit. The quantity of adapter ligated fragments was determined by qPCR using the KAPA SYBR FAST library quantification kit for Illumina GA (KAPA Biosystems).

Single read sequencing with 98 bases read length was performed using the HiSeq2000 system (Illumina) according to the manufacturer's protocols. Images were base called and quality filtered using the analysis pipe-line supplied together with the instrument. Sequencing was performed using the SNP&SEQ Technology Platform in Uppsala. Sequences were analyzed with CLC genomics workbench (CLC bio, Aarhus, Denmark) with analyses of SNP, DIP and low coverage regions.

### Growth rate measurements and competitions

Growth rates were measured in exponential phase in both LB and M9-media supplemented with 0.2% glycerol. Cells were grown overnight and diluted 1000-fold before added to a bioscreen plate in quadruplicates. OD_600_ was measured for 16 h at 37°C with continuous shaking at medium intensity in a Bioscreen C reader (Labsystems). Competitions were performed between strains carrying *galK*::CFP-*bla* and *galK*::YFP-*bla* on their chromosomes in both LB and M9-media supplemented with 0.2% glycerol. The competitors were grown separately overnight and then mixed together at 1∶1 ratio in fresh medium. At the same time cells were diluted in 1×PBS and after 1 h incubation at RT, approximately 100,000 cfu were counted in a FACS aria cell sorter to measure the fraction of YFP/CFP expressing cells respectively. Every day cells were diluted 1∶1000 in fresh media for continued competition and simultaneously in 1×PBS for FACS counting. Logarithmic ratios of YFP vs. CFP were plotted against number of generations and the growth advantage/disadvantage (Δs) of the mutant population was obtained from the slope of the curve.

### Evolution of wild-type *S. typhimurium* to faster growth in LB medium

Six independent lineages of wild type *S. typhimurium* were evolved for faster growth in LB medium for 1000 generations. Every day 1.5 ul culture (approximately 10^6^ cells) was transferred to 1.5 ml fresh LB medium and grown over night at 37°C, 200 rpm. Strains were frozen every 100- to 150 generations in LB supplemented with 10% DMSO at −80°C. After 1000 generations individual colonies from 3 of the 6 lineages and whole populations of the remaining 3 lineages were chosen for whole-genome sequencing. Whole-genome sequencing and DNA preparations were performed as described above (Mapping of exact deletion endpoints by whole-genome sequencing).

## Supporting Information

Figure S1 Intrinsic deletion rate per (kbp)^2^ per generation for each insertion position. Blue is the best estimate based on normalization by L1*_est_*×L2*_est_*; green shows the lower estimate based on 95% confidence, and red shows the upper estimate based on the assumption that the allowed deletion range equals that observed (i.e. L1 = M1, L2 = M2, which is the minimum possible). See Figure 1B for the actual locations of the different insertion positions.
(PDF)

Figure S2 Location and size of 30 deletions (numbered 1–30 to the left). Deleted material is indicated with a dashed line and remaining chromosomal material with a solid line. The deletometer is indicated as a rectangle where differences in shading indicate left (light) and right (dark) ends of the element. Numbers above the deletometer indicates nucleotide position from left to right within the deletometer (11,777 bp long) and numbers above solid line indicates nucleotide position in the chromosome.
(PDF)

Figure S3 Change in selection coefficient (Δs) for each mutant grown in single cultures in LB- versus M9-medium supplemented with glycerol (*A*) and for each mutant grown in single culture versus competitions in LB-medium (*B*).
(PDF)

Figure S4 Calculated take-over times to reach 50% of the population as a function of the deletion rate. Curves from Eq. (S4) in Text S1: *s* = 0.005 (blue) and *s* = 0.05 (red); *N* = 10^7^ (solid), *N* = 10^8^ (dashed), and *N* = 10^9^ (dotted). Circles and squares are from the stochastic model, Eqs. (S6)–(S8) in Text S1 with *N* = 10^7^ and *s* = 0.005 and 0.05, respectively.
(PDF)

Table S1 Characteristics of analyzed deletions.
(DOCX)

Table S2 Sequence changes identified in six serially passaged lineages of wild-type *S. typhimurium*.
(DOCX)

Table S3 Estimation of the fitness gain of loss of the deleted genes based on the fraction of total amino acid content.
(DOCX)

Table S4 Strains of *S. typhimurium* used in this study.
(DOCX)

Table S5 Primers used in this study.
(DOCX)

Text S1 Contains supporting materials and methods.
(PDF)
