# Supplementary figures and images for: Selection-Driven Gene Loss in Bacteria
Source: PLoS Genet. 2012 Jun 28;8(6):e1002787. doi: 10.1371/journal.pgen.1002787 (PMC3386194; doi:10.1371/journal.pgen.1002787)

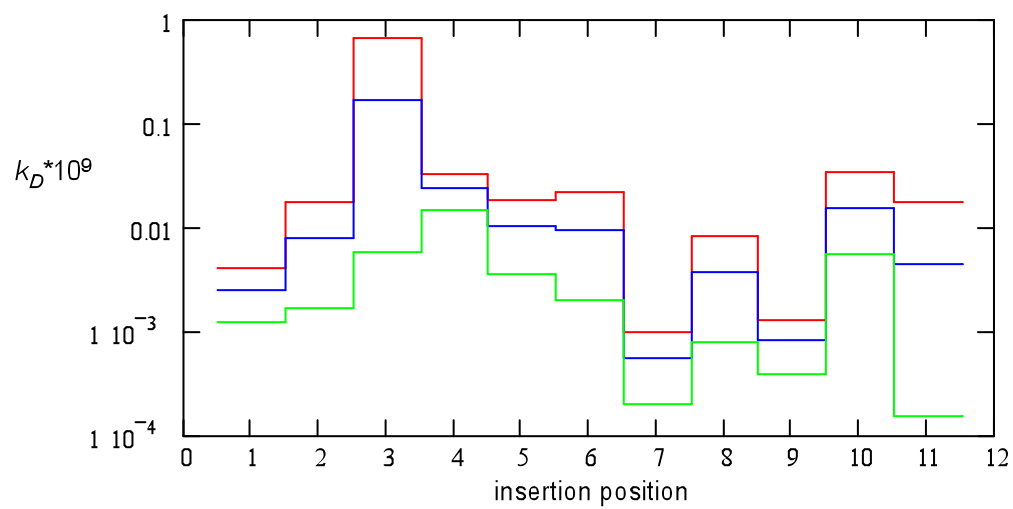

Supplement: Figure S1 — Intrinsic deletion rate per (kbp)2 per generation for each insertion position. Blue is the best estimate based on normalization by L1est×L2est; green shows the lower estimate based on 95% confidence, and red shows the upper estimate based on the assumption that the allowed deletion range equals that observed (i.e. L1 = M1, L2 = M2, which is the minimum possible). See Figure 1B for the actual locations of the different insertion positions. (PDF) [file pgen.1002787.s001.pdf]

Tn10 construct

0 bp 11777 bp

TL TR

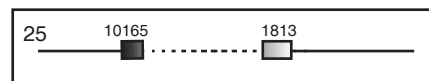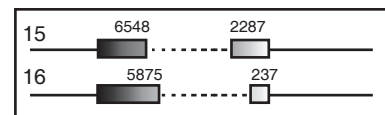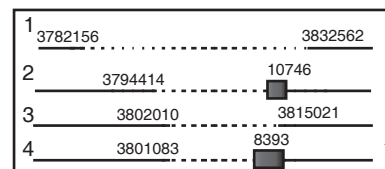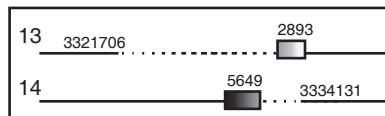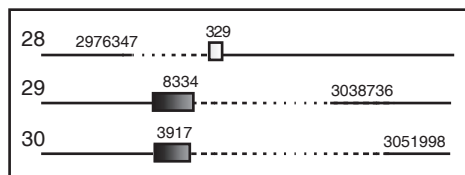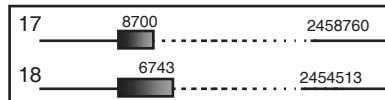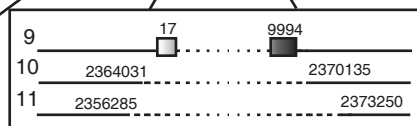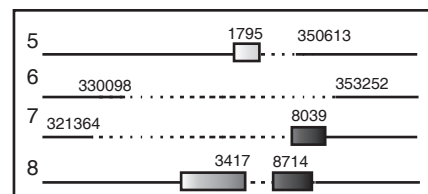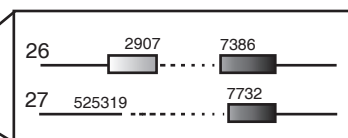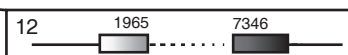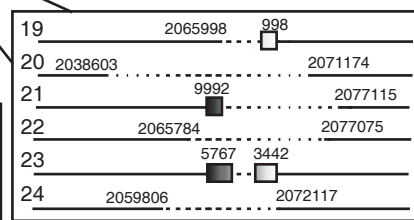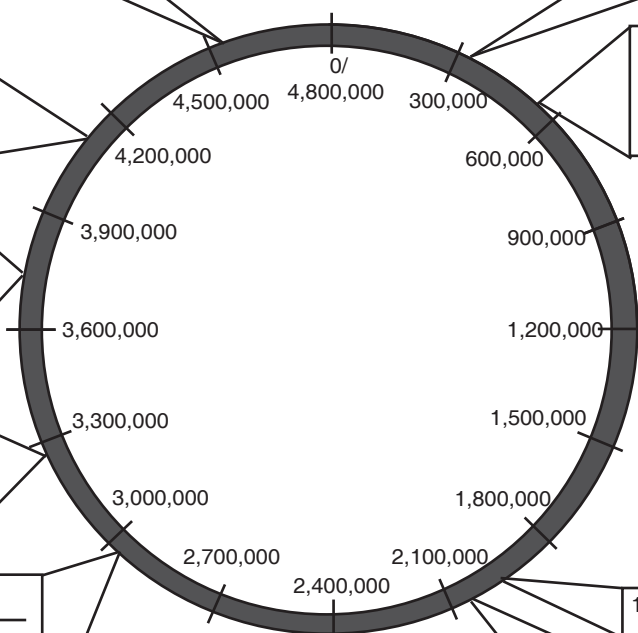

Supplement: Figure S2 — Location and size of 30 deletions (numbered 1–30 to the left). Deleted material is indicated with a dashed line and remaining chromosomal material with a solid line. The deletometer is indicated as a rectangle where differences in shading indicate left (light) and right (dark) ends of the element. Numbers above the deletometer indicates nucleotide position from left to right within the deletometer (11,777 bp long) and numbers above solid line indicates nucleotide position in the chromosome. (PDF) [file pgen.1002787.s002.pdf]

**A**

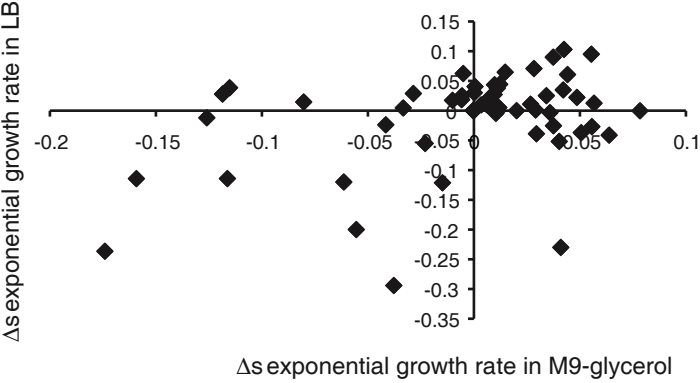

**B**

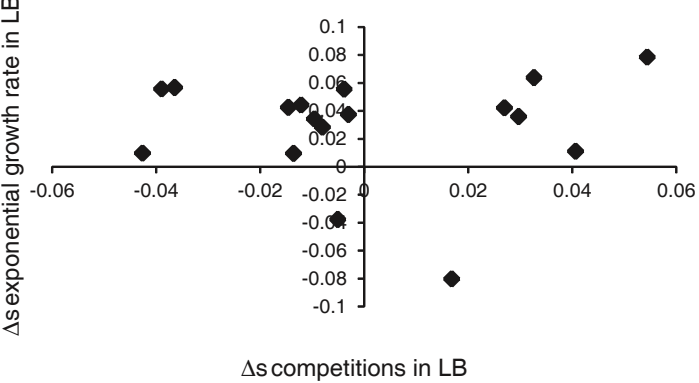

Supplement: Figure S3 — Change in selection coefficient (Δs) for each mutant grown in single cultures in LB- versus M9-medium supplemented with glycerol (A) and for each mutant grown in single culture versus competitions in LB-medium (B). (PDF) [file pgen.1002787.s003.pdf]

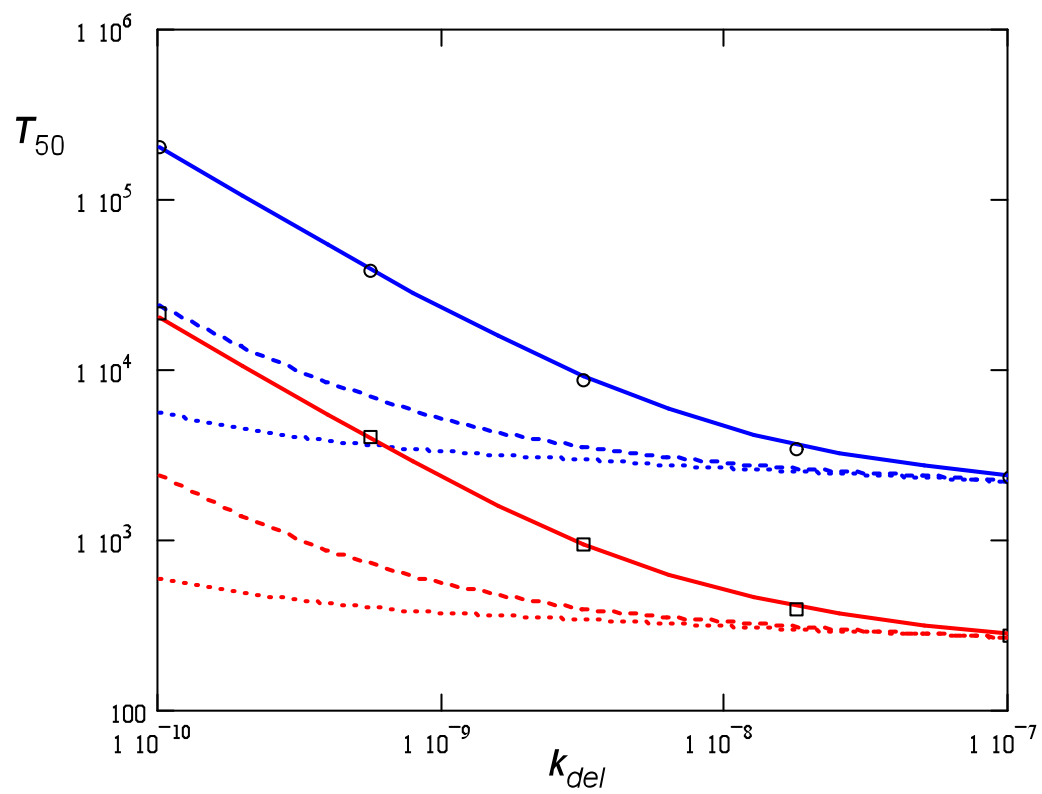

Supplement: Figure S4 — Calculated take-over times to reach 50% of the population as a function of the deletion rate. Curves from Eq. (S4) in Text S1: s = 0.005 (blue) and s = 0.05 (red); N = 107 (solid), N = 108 (dashed), and N = 109 (dotted). Circles and squares are from the stochastic model, Eqs. (S6)–(S8) in Text S1 with N = 107 and s = 0.005 and 0.05, respectively. (PDF) [file pgen.1002787.s004.pdf]
