# Supplementary material for: Selection-Driven Gene Loss in Bacteria
Source: PLoS Genet. 2012 Jun 28;8(6):e1002787. doi: 10.1371/journal.pgen.1002787 (PMC3386194; doi:10.1371/journal.pgen.1002787)
Supplement: Table S1 — Characteristics of analyzed deletions. (DOCX) [file pgen.1002787.s005.docx]

**Table S1.** Characteristics of analyzed deletions

| Strain | | Deletion endpoints | Deletion size | Sequence left of deletometer | Homology  (at endpoints) | Fitness as relative  growth rate  (in exponential phase) | | Fitness as  selection coefficient  (in competitions against parental wt) |
| --- | --- | --- | --- | --- | --- | --- | --- | --- |
| Parental strain: **DA16821**  Orientation/Location:  **TL-TR/3802298** | |  |  |  |  | LB | M9-gly | LB |
| 1 | DA16903 | 3782156-3832562bp | 62183 bp | 0bp | 10bp  TCGCTTCCCG | 1.01±0.04 | 1.04±0.00 | -0.043±0.0088 |
| 2 | DA16904 | 3794414bp (on chrom)  -10746bp (in deletometer) | 18630 bp | 1032bp | 12bp  GGTCATCCAGCG | 1.01±0.02 | 1.01±0.01 | -0.014±0.0077 |
| 3 | DA16905 | 3802010-3815021bp | 24788 bp | 0bp | 15bp (-3)  CAGG(C/T)TTT  (C/T)CG(T/C)CGC | 0.83±0.01 | 0.76±0.02 | n.d |
| 4 | DA16907 | 3801083bp (on chrom)  -8393bp (in deletometer) | 9608bp | 3384bp | 1bp  G | 0.92±0.02 | 1.02±0.07 | 0.017±0.009 |
| Parental strain: **DA16823**  Orientation/Location:  **TL-TR/349874** | |  |  |  |  |  |  |  |
| 5 | DA16906 | 1795bp (in deletometer)  -350613bp (on chrom) | 10721 bp | 1795bp | 8bp  TTTTCATT | 0.96±0.03 | 0.75±0.1 | -0.005±0.016 |
| 6 | DA16913 | 330098-353252bp | 34931 bp | 0bp | 12bp  CAGGCTGCCGCC | 1.06±0.02 | 1.1±0.02 | -0.004±0.0081 |
| 7 | DA16916 | 321364bp (on chrom)  -8039bp (in deletometer) | 36549bp | 3738bp | 11bp  CTGGATGCGGC | 1.04±0.03 | 1.06±0.05 | -0.012±0.0071 |
| 8 | DA16917 | Deletion in deletometer  3417-8714bp | 5297bp | 6480bp | 4bp  GTAG | 1.04±0.01 | 1.1±0.02 | -0.015±0.004 |
| Parental strain: **DA16828**  Orientation/Location:  **TR-TL 2366992** | |  |  |  |  |  |  |  |
| 9 | DA16920 | Deletion in deletometer  17-9994bp | 9977bp | 1800bp | 1bp  T | 1.02±0.04 | 1.09±0.01 | n.d |
| 10 | DA16925 | 2364031-2370135bp | 17881bp | 0bp | 4bp (-1)  AAA(T/C) | 0.95±0.01 | 0.80±0.00 | n.d |
| 11 | DA16927 | 2356285-2373250bp | 28742bp | 0bp | 13bp  CGCGCCAGGCGGA | 0.94±0.01 | 0.88±0.00 | n.d |
| Parental strain: **DA16829**  Orientation/Location:  **TR-TL 1924601** | |  |  |  |  |  |  |  |
| 12 | DA16934 | Deletion in deletometer  1965-7346bp | 5381bp | 6396bp | 0bp | 1.07±0.03 | 1.04±0.05 | n.d |
| Parental strain: **DA16831**  Orientation/Location:  **TR-TL 3334101** | |  |  |  |  |  |  |  |
| 13 | DA16938 | 3321706bp (on chrom)  -2893bp (in deletometer) | 21279bp | 2893bp | 12bp  GGGAAAACGCCT | 1.04±0.05 | 0.77±0.00 | n.d |
| 14 | DA16940 | 5649bp (in deletometer)  -3334131bp (on chrom) | 5679bp | 6128bp | 12bp  AGCGGCCCCGGC | 0.96±0.04 | 0.98±0.05 | n.d |
| Parental strain: **DA16833**  Orientation/Location:  **TR-TL 4066253** | |  |  |  |  |  |  |  |
| 15 | DA16945 | Deletion in deletometer  2287-6548bp | 4261bp | 7516bp | 12bp (-3)  C(G/C)GCG(C/A)  AA(A/T) | 1.04±0.03 | 0.94±0.01 | n.d |
| 16 | DA16950 | Deletion in deletometer  237-5875bp | 5638bp | 6139bp | 6bp  AGGTGG | 1.06±0.03 | 0.99±0.06 | n.d |
| Parental strain: **DA16836**  Orientation/Location:  **TR-TL 2453577** | |  |  |  |  |  |  |  |
| 17 | DA16956 | 8700bp (in deletometer)  -2458760bp (on chrom) | 13883bp | 3077bp | 1bp  T | 1.04±0.01 | 0.97±0.07 | n.d |
| 18 | DA16961 | 6743bp (in deletometer)  -2454513bp (on chrom) | 7679bp | 5034bp | 1bp  T | 1.05±0.01 | 0.94±0.09 | n.d |
| Parental strain: **DA16837**  Orientation/Location:  **TR-TL 2066286** | |  |  |  |  |  |  |  |
| 19 | DA16954 | 2065998bp (on chrom)  -998bp (in deletometer) | 11067bp | 998bp | 2bp  TG | 1.06±0.02 | 0.96±0.04 | n.d |
| 20 | DA16963 | 2038603-2071174bp | 44348bp | 0bp | 13bp (-1)  AGCCAGC(G/T)GCGCC | 1.08±0.00 | 0.98±0.02 | 0.054±0.009 |
| 21 | DA16965 | 9992bp (in deletometer)  -2077115bp (on chrom) | 20821bp | 1785bp | 1bp  G | 1.06±0.02 | 0.97±0.06 | 0.033±0.005 |
| 22 | DA16966 | 2065784-2077075bp | 23068bp | 0bp | 3bp  CTG | 1.04±0.02 | 1.0±0.04 | 0.027±0.005 |
| 23 | DA16967 | Deletion in deletometer  3442-5767bp | 2325bp | 9452bp | 9bp  AAATCGTCG | 1.04±0.01 | 0.95±0.08 | 0.03±0.002 |
| 24 | DA16969 | 2059806-2072117bp | 24088bp | 0bp | 1bp  A | 1.01±0.01 | 1.0±0.05 | 0.041±0.005 |
| Parental strain: **DA16838**  Orientation/Location:  **TR-TL 4589954** | |  |  |  |  |  |  |  |
| 25 | DA16974 | Deletion in deletometer  1813-10165bp | 8352bp | 3425bp | 15bp (-2)  TCCTTT(A/-)CAC  (T/-)CAGT | 1.03±0.03 | 0.99±0.06 | -0.010±0.002 |
| Parental strain: **DA16839**  Orientation/Location:  **TL-TR 526690** | |  |  |  |  |  |  |  |
| 26 | DA16981 | Deletion in deletometer  2907-7386bp | 4479bp | 7298bp | 10bp  GTTTTCCCCT | 1.01±0.02 | 0.99±0.04 | n.d |
| 27 | DA16983 | 525319bp (on chrom)  -7732bp(in deletometer) | 9103bp | 4045bp | 11bp (-2)  G(T/C)GGCGG  GG(C/A)G | 1.01±0.02 | 1.02±0.03 | n.d |
| Parental strain: **DA16840**  Orientation/Location:  **TR-TL 2992238** | |  |  |  |  |  |  |  |
| 28 | DA16984 | 2976347bp (on chrom)  -329bp (in deletometer) | 27339bp | 329bp | 6bp (-1)  CAT(A/T)AC | 1.0±0.01 | 1.01±0.04 | n.d |
| 29 | DA16985 | 8334bp (in deletometer  -3038736bp (in chrom) | 54832bp | 3443pb | 7bp  ACGCGCC | 0.99±0.00 | 0.98±0.06 | n.d |
| 30 | DA16986 | 3917bp (in deletometer  -3051998bp (in chrom) | 63677bp | 7860bp | 16bp (-4)  GCCG(G/A)AG  (G/C)(A/)GCGCC  (G/T)GG | 0.99±0.01 | 0.98±0.02 | n.d |
