# Supplementary material for: Selection-Driven Gene Loss in Bacteria
Source: PLoS Genet. 2012 Jun 28;8(6):e1002787. doi: 10.1371/journal.pgen.1002787 (PMC3386194; doi:10.1371/journal.pgen.1002787)
Supplement: Table S2 — Sequence changes identified in six serially passaged lineages of wild-type S. typhimurium. (DOCX) [file pgen.1002787.s006.docx]

**Table S2.** Sequence changes identified in six serially passaged lineages of wild type *S. typhimurium*.

| Strain | mutations | deletions |
| --- | --- | --- |
| 20840 | flhC(Pro58Leu) | uvrC-yecS |
|  | rfbP(Arg80Cys) | *manY*-STM1836 |
|  | rfbV(-1 fs) |  |
|  |  |  |
| 20841 | ftsW(Lys370Thr) |  |
|  | fimZ(Ile120Val) |  |
|  | STM0893(Ala381Ser) |  |
|  | STM0898(Val215Ile) |  |
|  | glpT(Gly382Ala) |  |
|  |  |  |
|  | Common between 20842 and 20843 |  |
| 20842 | barA(Ser501Pro) | fliG(54bp) |
|  | rfaK(Thr10Ala) |  |
|  | pyrB(Ala459Val) |  |
|  | crl(Arg32fs) |  |
|  |  |  |
|  | common for 20843, 20844 and 20845 |  |
|  | common for 20843 and 20844 |  |
|  | common for 20843 and 20845 |  |
| 20843 | leuA | STM1760 |
|  | cueO(Val434Ala) |  |
|  | allB(Pro442Ser) |  |
|  | STM0716(Ala146Thr) |  |
|  | STM0769(Leu180Phe) |  |
|  | sseL(Leu165Pro) |  |
|  | ypfG(Asp205Gly) |  |
|  | mltB(Ile152Asn) |  |
|  | ygfX(Thr62Ala) |  |
|  | deaD(Thr83Ala) |  |
|  | ompR(Val203Ala) |  |
|  | glgC(Phe259Ser) |  |
|  | hemE(Arg28Gln) |  |
|  | srfJ(Pro147Leu) |  |
|  | STM4427 |  |
|  | yjgP(Thr216Ala) |  |
|  | STM0769(Leu179fs) |  |
|  | STM0769(Gly183fs) |  |
|  | crl(Arg32fs) |  |
|  | STM2729 |  |
|  | rrsD |  |
|  | STM4067(His2Arg) |  |
|  | flgF(Met9fs) |  |
|  | yigC |  |
|  | fdoG |  |
|  | yihZ |  |
|  | ompC(tyr348fs) |  |
|  | barA(Leu185fs) |  |
|  | carA(Asn240Ser) |  |
|  | yafS(Ala79Val) |  |
|  | dcuC(Gly29Asp) |  |
|  | ycaD(Thr375Ala) |  |
|  | sifB(Ser133Pro) |  |
|  | rnb(Trp234*) |  |
|  | fliQ(Leu70Pro) |  |
|  | yejE |  |
|  | mutH(Lys79Gln) |  |
|  | STM3035 |  |
|  | parC(Pro542Leu) |  |
|  | yhcG |  |
|  | aroE(Thr220Ala) |  |
|  | atpD(Ile55Val) |  |
|  | uvrD(Pro568Leu) |  |
|  | yigC(Thr427Ala) |  |
|  | ushB(Ala10Val) |  |
|  | ulaA(Thr48Ala) |  |
|  | rfaK(Gly310fs) |  |
|  | glpF(Val52fs) |  |
|  |  |  |
|  | common for 20843, 20844 and 20845 |  |
|  | common for 20843 and 20845 |  |
|  | common for 20844 and 20845 |  |
| 20844 | ompC(Tyr321Asp) | STM1559-narZ |
|  | ompR(Arg210Cys) | OmpC |
|  | rfaJ(Pro269Leu) |  |
|  | fliQ(Leu70Pro) |  |
|  | STM2729 |  |
|  | carA(Asn240Ser) |  |
|  | yafS(Ala79Val) |  |
|  | dcuC(Gly29Asp) |  |
|  | ycaD(Thr375Ala) |  |
|  | sifB(Ser133Pro) |  |
|  | rnb(Trp234*) |  |
|  | fliQ(Leu70Pro) |  |
|  | yejE |  |
|  | STM2729 |  |
|  | mutH(Lys79Gln) |  |
|  | STM3035 |  |
|  | parC(Pro542Leu) |  |
|  | yhcG |  |
|  | aroE(Thr220Ala) |  |
|  | atpD(Ile55Val) |  |
|  | uvrD(Pro568Leu) |  |
|  | yigC(Thr427Ala) |  |
|  | ushB(Ala10Val) |  |
|  | ulaA(Thr48Ala) |  |
|  | flgF(Met9fs) |  |
|  | yigC |  |
|  | fdoG |  |
|  | yihZ |  |
|  | ompC(tyr348fs) |  |
|  | barA(Leu185fs) |  |
|  | rfaK(Gly310fs) |  |
|  | glpF(Val52fs) |  |
|  |  |  |
|  | common for 20843, 20844 and 20845 |  |
|  | common for 20843 and 20844 |  |
|  | common for 20844 and 20845 |  |
| 20845 | bcfH(Asn231Ser) |  |
|  | yabI(val32Ala) |  |
|  | ftsI(Leu444Pro) |  |
|  | STM0201 |  |
|  | rrlH |  |
|  | yafC(Gly93Asp) |  |
|  | prpR(Leu502Ser) |  |
|  | dpiB |  |
|  | STM0692 |  |
|  | galE(Gly10Ser) |  |
|  | bioA(val38Ala) |  |
|  | lonH(Val544Ala) |  |
|  | putA |  |
|  | potA |  |
|  | selD(Ala132Val) |  |
|  | ydiJ(Thr559Ala) |  |
|  | ssaP(Ser16Ala) |  |
|  | ydhE |  |
|  | STM1441(Val575Ala) |  |
|  | STM1527(Val26Ala) |  |
|  | nmpC(Ser291Pro) |  |
|  | rnb(Trp234*) |  |
|  | araH |  |
|  | cbiL(Lys92Arg) |  |
|  | rplY(Val97Ala) |  |
|  | ompC(Gln307*) |  |
|  | rcsC(Thr483Ala) |  |
|  | ppk |  |
|  | shdA(Asn355Ser) |  |
|  | iroB(Val216Ala) |  |
|  | alaS(Thr674Ala) |  |
|  | STM2943 |  |
|  | pyrG |  |
|  | gvcP(Asn432Ser) |  |
|  | yggN(Asn45Ser) |  |
|  | ygiK |  |
|  | yqiB |  |
|  | fpkA |  |
|  | mrcA |  |
|  | yhgE(Arg201Cys) |  |
|  | envZ(Tyr31Cys) |  |
|  | STM23633 |  |
|  | yieN |  |
|  | STM3942 |  |
|  | yiiR(Thr133Ile) |  |
|  | malG(Phe223Leu) |  |
|  | STM4445(Asp220Gly) |  |
|  | STM4489 |  |
|  | foxA(Pro518fs) |  |
|  | fumC(Trp22fs) |  |
|  | yfcA(Pro161fs) |  |
|  | ratB(Arg1344fs) |  |
|  | ptsP(Gly742fs) |  |
|  | STM3133(Ala270fs) |  |
|  | STM3350(Glu202fs) |  |
|  | rrsD |  |
|  | STM4067(His2Arg) |  |
|  | flgF(Met9fs) |  |
|  | yigC |  |
|  | fdoG |  |
|  | yihZ |  |
|  | ompC(tyr348fs) |  |
|  | barA(Leu185fs) |  |
|  | fliQ(Leu70Pro) |  |
|  | carA(Asn240Ser) |  |
|  | yafS(Ala79Val) |  |
|  | dcuC(Gly29Asp) |  |
|  | ycaD(Thr375Ala) |  |
|  | sifB(Ser133Pro) |  |
|  | rnb(Trp234*) |  |
|  | fliQ(Leu70Pro) |  |
|  | yejE |  |
|  | STM2729 |  |
|  | mutH(Lys79Gln) |  |
|  | STM3035 |  |
|  | parC(Pro542Leu) |  |
|  | yhcG |  |
|  | aroE(Thr220Ala) |  |
|  | atpD(Ile55Val) |  |
|  | uvrD(Pro568Leu) |  |
|  | yigC(Thr427Ala) |  |
|  | ushB(Ala10Val) |  |
|  | ulaA(Thr48Ala) |  |
|  | flgF(Met9fs) |  |
|  | yigC |  |
|  | fdoG |  |
|  | yihZ |  |
|  | ompC(tyr348fs) |  |
|  | barA(Leu185fs) |  |
|  | rfaK(Gly310fs) |  |
|  | glpF(Val52fs) |  |
