# Supplementary material for: Selection-Driven Gene Loss in Bacteria
Source: PLoS Genet. 2012 Jun 28;8(6):e1002787. doi: 10.1371/journal.pgen.1002787 (PMC3386194; doi:10.1371/journal.pgen.1002787)
Supplement: Table S3 — Estimation of the fitness gain of loss of the deleted genes based on the fraction of total amino acid content. (DOCX) [file pgen.1002787.s007.docx]

**Table S3.** Estimation of the fitness gain of loss of the deleted genes based on the fraction of total amino acid content.

| **Genes included in deletion** | **Fraction of total amino acid content in cell** | **Expected gain** | **Observed gain** |
| --- | --- | --- | --- |
| fliG (from evolution experiment) |  |  |  |
| STM1970 | 0.00033 |  |  |
| Sum | 0.00033 | 0.033% | 3.2±0.56% |
| uvrC-yecS (from evolution experiment) |  |  |  |
| STM1946 | 4.13E-05 |  |  |
| STM1947 | 2.96E-05 |  |  |
| STM1949 | 7.88E-05 |  |  |
| STM1950 |  |  |  |
| STM1951 | 0.00011 |  |  |
| STM1952 | 1.183E-05 |  |  |
| Sum | 0.00027 | 0.027% | 4.7±0.71% |
| fliQ-STM1994(from deletometer) |  |  |  |
| STM1980 |  |  |  |
| STM1981 |  |  |  |
| STM1982 |  |  |  |
| STM1983 |  |  |  |
| STM1984 | 2.56E-05 |  |  |
| STM1985 |  |  |  |
| STM1986 | 1.381E-05 |  |  |
| STM1987 |  |  |  |
| STM1988 | 1.18E-05 |  |  |
| STM1989 | 1.77E-05 |  |  |
| STM1990 |  |  |  |
| STM1991 |  |  |  |
| STM1992 | 2.96E-05 |  |  |
| STM1993 |  |  |  |
| STM1994 |  |  |  |
| Sum | 9.86E-05 | 0.0099% | 3.3±0.5% |
| uvrY-STM1987(from deletometer) |  |  |  |
| STM1947 | 2.96E-05 |  |  |
| STM1948 |  |  |  |
| STM1949 | 7.88E-05 |  |  |
| STM1950 |  |  |  |
| STM1951 | 0.00011 |  |  |
| STM1952 | 1.18E-05 |  |  |
| STM1953 | 0.00027 |  |  |
| STM1954 | 0.00031 |  |  |
| STM1955 | 0.00015 |  |  |
| STM1956 | 0.00017 |  |  |
| STM1957 |  |  |  |
| STM1958 | 0.00032 |  |  |
| STM1959 | 0.0076 |  |  |
| STM1960 | 0.00067 |  |  |
| STM1961 | 0.00010 |  |  |
| STM1962 | 6.11E-05 |  |  |
| STM1963 |  |  |  |
| STM1964 | 0.00019 |  |  |
| STM1965 |  |  |  |
| STM1966 |  |  |  |
| STM1967 |  |  |  |
| STM1968 | 2.76E-05 |  |  |
| STM1969 | 0.00033 |  |  |
| STM1970 | 0.00033 |  |  |
| STM1971 | 0.00016 |  |  |
| STM1972 | 0.00021 |  |  |
| STM1973 | 1.18E-05 |  |  |
| STM1974 | 3.75E-05 |  |  |
| STM1975 | 0.00015 |  |  |
| STM1976 | 0.00030 |  |  |
| STM1977 | 0.00014 |  |  |
| STM1978 |  |  |  |
| STM1979 |  |  |  |
| STM1980 |  |  |  |
| STM1981 |  |  |  |
| STM1982 |  |  |  |
| STM1983 |  |  |  |
| STM1984 | 2.56E-05 |  |  |
| STM1985 |  |  |  |
| STM1986 | 1.38E-05 |  |  |
| STM1987 |  |  |  |
| Sum | 0.012 | 1.2% | 5.4±0.9% |
| fliP-STM1994(from deletometer) |  |  |  |
| STM1979 |  |  |  |
| STM1980 |  |  |  |
| STM1981 |  |  |  |
| STM1982 |  |  |  |
| STM1983 |  |  |  |
| STM1984 | 2.56E-05 |  |  |
| STM1985 |  |  |  |
| STM1986 | 1.38E-05 |  |  |
| STM1987 |  |  |  |
| STM1988 | 1.18E-05 |  |  |
| STM1989 | 1.77E-05 |  |  |
| STM1990 |  |  |  |
| STM1991 |  |  |  |
| STM1992 | 2.96E-05 |  |  |
| STM1993 |  |  |  |
| STM1994 |  |  |  |
| Sum | 9.86E-05 | 0.0099% | 2.7±0.5% |
| fliH-yedI(from deletometer) |  |  |  |
| STM1971 | 0.00016 |  |  |
| STM1972 | 0.00021 |  |  |
| STM1973 | 1.18E-05 |  |  |
| STM1974 | 3.75E-05 |  |  |
| STM1975 | 0.00015 |  |  |
| STM1976 | 0.00030 |  |  |
| STM1977 | 0.00014 |  |  |
| STM1978 |  |  |  |
| STM1979 |  |  |  |
| STM1980 |  |  |  |
| STM1981 |  |  |  |
| STM1982 |  |  |  |
| STM1983 |  |  |  |
| STM1984 | 2.56E-05 |  |  |
| STM1985 | 0 |  |  |
| STM1986 | 1.38E-05 |  |  |
| STM1987 |  |  |  |
| STM1988 | 1.18E-05 |  |  |
| STM1989 | 1.77E-05 |  |  |
| Sum | 0.0011 | 0.11% | 4.1±0.5% |
| yhjL-bcsA(from deletometer) |  |  |  |
| STM3616 | 9.46E-05 |  |  |
| STM3617 | 1.18E-05 |  |  |
| STM3618 | 0.00038 |  |  |
| STM3619 |  |  |  |
| Sum | 0.00049 | 0.049% | 0.5±0.12% |
