# Supplementary material for: Selection-Driven Gene Loss in Bacteria
Source: PLoS Genet. 2012 Jun 28;8(6):e1002787. doi: 10.1371/journal.pgen.1002787 (PMC3386194; doi:10.1371/journal.pgen.1002787)
Supplement: Table S4 — Strains of S. typhimurium used in this study. (DOCX) [file pgen.1002787.s008.docx]

**Table S4.** Strains of *S. typhimurium* used in this study.

Designation Relevant genotype Origin

DA2248 /F’128, *pro*+, *lac*+, *zzf*1837::Tn10dtet lab collection

DA2249 /pNK974 (transposase plasmid) lab collection

DA6192 LT2 wild type lab collection

DA14551 NEB /pSK07 this work

DA15110 *galK*::YFP-*bla* lab collection

DA15111 *galK*::CFP-*bla* lab collection

DA16817 *metA*22, *metE*551, *trpD*2, *ilv*-452, *leu*-, this work

*pro*- (leaky), *hsdLT*6, *hsdSA*29, *hsdB*,

*moaA*::kan(sw) /F’128, *pro*+, *lac*::rif(sw)

*zzf*1837::deletometer

DA16821 DEL*moaA*, *bcsA*::deletometer this work

DA16823 DEL*moaA*, STM0305::deletometer this work

DA16828 DEL*moaA*, *micF*::deletometer this work

DA16829 DEL*moaA*, *sdaA*::deletometer this work

DA16831 DEL*moaA*, *ygiK*::deletometer this work

DA16833 DEL*moaA*, *aroE*::deletometer this work

DA16836 DEL*moaA*, STM2340::deletometer this work

DA16837 DEL*moaA*, *fliQ*::deletometer this work

DA16838 DEL*moaA*, *yjeP*::deletometer this work

DA16839 DEL*moaA*, *rpmJ2*::deletometer this work

DA16840 DEL*moaA*, *hycH*::deletometer this work

DA16903 DEL*moaA*, DEL*yhjD-bisC* this work

DA16904 DEL*moaA*, DEL*yhjL-bcsA* this work

DA16905 DEL*moaA*, DEL*bcsA-dppA* this work

DA16906 DEL*moaA*, DELSTM0305-STM0306 this work

DA16907 DELmoaA, DEL*bcsA* this work

DA16913 DEL*moaA*, DELSTM0289-*fadE* this work

DA16916 DEL*moaA*, DELSTM0282-STM0306 this work

DA16917 DEL*moaA*, STM0305::deletometer, this work

DEL3417-8714bp

DA16920 DEL*moaA*, *micF*::deletometer DEL17-9994bp this work

DA16925 DEL*moaA*, DEL*ada-rcsB* this work

DA16927 DEL*moaA*, DEL*napG-rcsC* this work

DA16934 DEL*moaA*, *sdaA*::deletometer, DEL1965-7346bp this work

DA16938 DEL*moaA*, DEL*metC-ygiK* this work

DA16940 DEL*moaA*, DEL*ygiK-sufI* this work

DA16945 DEL*moaA*, *aroE*::deletometer, DEL2287-6548bp this work

DA16950 DEL*moaA*, *aroE*::deletometer, DEL237-5875bp this work

DA16954 DEL*moaA*, DEL*fliP-fliQ* this work

DA16956 DEL*moaA*, DELSTM2340-STM2346 this work

DA16961 DEL*moaA*, DELSTM2340-STM2341 this work

DA16963 DEL*moaA*, DEL*uvrY*-STM1987 this work

DA16965 DEL*moaA*, DEL*fliQ*-STM1994 this work

DA16966 DEL*moaA*, DEL*fliP*-STM1994 this work

DA16967 DEL*moaA*, *fliQ*::deletometer, DEL3442-5767 this work

DA16969 DEL*moaA*, *fliH-yedI* this work

DA16974 DEL*moaA*, *yjeP*::deletometer, DEL1813-10165bp this work

DA16981 DEL*moaA*, *rpmJ*2::deletometer, DEL2907-7386bp this work

DA16983 DEL*moaA*, DEL*ylaB-rpmJ*2 this work

DA16984 DEL*moaA*, DEL*ygaD-hycH* this work

DA16985 DEL*moaA*, DEL*hycH-invA* this work

DA16986 DEL*moaA*, DEL*hycH-mutS* this work

DA20840 LT2, evolved 1000generations in LB, lineage 1 this work

DA20841 LT2, evolved 1000generations in LB, lineage 2 this work

DA20842 LT2, evolved 1000generations in LB, lineage 3 this work

DA20843 LT2, evolved 1000generations in LB, lineage 4 this work

DA20844 LT2, evolved 1000generations in LB, lineage 5 this work

DA20845 LT2, evolved 1000generations in LB, lineage 6 this work

Plasmids

pSK07 pBAD30::*moaA*-cam this work
