# Supplementary material for: Selection-Driven Gene Loss in Bacteria
Source: PLoS Genet. 2012 Jun 28;8(6):e1002787. doi: 10.1371/journal.pgen.1002787 (PMC3386194; doi:10.1371/journal.pgen.1002787)
Supplement: Table S5 — Primers used in this study. (DOCX) [file pgen.1002787.s009.docx]

**Table S5.** Primers used in this study

Moa_KO_F: ggaagaaatgactccgcctcccgtatttggaaaggtgtactgtaggctggagctgcttc

MoaA_KO_R: cagcgctgacctgactcatctgaaatctccttattaaggacatatgaatatcctcctta

moaAextF (hindIII): gcgcaagcttataagcagcgctacagtagc

moaAR (smaI): atatgggcccttaaccgccaatgtaagata

pBAD30F: ttagcggatcctacctgacg

pBAD30R: actcagaagtgaaacgccgt

del_verificationF: aggctttctcttttttcaa

del_verificationR: gcgccttatccgaccaacat

deleto_insF: aaaacctatctattttatttatctttcaagctcaataaaaagccgcggtaatgcggctaatgtagatcgc

deleto_insR: cgaaaaacctaaaagagcttgccgataaaaaaggccaatttattgctattgaatcgcaggctattctggt

tn10ins_verF: cgcgaaataaacgaccggga

tn10ins_verR: ctgcgacatcgtataacgtta
