# Supplementary material for: Selection-Driven Gene Loss in Bacteria
Source: PLoS Genet. 2012 Jun 28;8(6):e1002787. doi: 10.1371/journal.pgen.1002787 (PMC3386194; doi:10.1371/journal.pgen.1002787)
Supplement: Text S1 — Contains supporting materials and methods. (PDF) [file pgen.1002787.s010.pdf]

Supporting Text S1

Construction of the transposon: In this work we measured spontaneous deletion rates at different locations on the bacterial chromosome using the same genetic screen. To allow this, we constructed a transposable element carrying a counter selectable marker, *moaA*, and the *lac* operon. In *S. typhimurium* LT2 inactivation of the *moaA* gene located on the chromosome makes cells chlorate resistant [1]. In earlier studies we have used this gene together with the adjacent galactose syntethesizing *gal* operon to study spontaneous deletion formation on the bacterial chromosome [2,3].

Construction of the pSK07 plasmid: First of all *moaA* and a gene encoding Cam resistance were amplified with PCR from the *S. typhimurium* chromosome and pKD3 respectively, with primers containing restriction sites at their ends (Table S5). Primers were constructed so that the amplified fragments would contain the genes own promoter respectively. The two PCR products were cut with hindIII restriction enzyme (Fermentas) for 2h at 37ºC and ligated together using T4 DNA ligase (Fermentas) at 16ºC o/n. The ligated product was run on a 1% SeaKem LE agarose gel (In Vitro) and a band of the correct size was purified using the GFX PCR and gel purification kit (GE healthcare). The pBAD30 plasmid and the purified ligated PCR product were cut with SmaI and XbaI restriction enzymes for 2h at 37ºC and ligated together with T4 DNA ligase at 16ºC o/n. The ligated product was transformed into New England Biolabs (NEB) high efficiency chemically competent cells by heat shock as described by the manufacturer (In Vitro). Transformants were selected on agar plates containing 100mg/L ampicillin over night at 37ºC and colonies were picked randomly and restreaked on 100mg/L ampicillin. Transformants were screened by colony PCR with pBAD30 primers (Table S5) and plasmids with inserts of the correct size were purified using the E.Z.N.A plasmid purification kit (Omega bio-tek). Plasmids were verified by sequencing with pBAD30 primers. The plasmid was verified phenotypically by insertion of the plasmid into a strain lacking *moaA* on the chromosome and screening for chlorate resistance.

The *moaA-cam* construct was amplified with PCR from the pSK07 plasmid using linear transformation primers (Table S5) and inserted into the *lacA* gene of the *lac*-operon found on *mudJ* transposon using linear transformation [4]. In short, cells containing a plasmid (pKD46) with the lamda red genes (*gam, bet, exo*) expressed

from an arabinose inducible promoter, were grown o/n in the presence of ampicillin. Cells were diluted 1:100 and ice for 30min. The cells were washed 3 times in 10% ice-cold glycerol and after the last wash the cells were resuspended in 200µl 10% glycerol. Linear DNA was electroporated into the cells using a Gene Pulser system from Bio-Rad (2.5kV, 200Ω, 25µF). Transformants were plated on LA-plates containing 20mg/L chloramphenicol and allowed to grow overnight. Transformants were picked at random and restreaked on LA-plates containing 20mg/L chloramphenicol. PCR with del_verification primers (Table S5) was used to verify linear-transformants. The whole *lac*-operon containing the *moaA-cam* construct in the *lacA* gene was amplified with linear transformation primers (Table S5). This linear DNA was ~8kb length and a long range PCR kit from Roche was used to amplify the DNA according to the manufacturer (Roche). DNA was purified using the Fermentas PCR purification kit and used for linear transformation grown for 1h before induction with 0.2% L-arabinose. Cells were grown to OD_{600} ~0.5 and put on as described above. The linear DNA was inserted into the non-coding region at the right end of the transposable element Tn10dtet. Transformants were verified with PCR using tn10ins_verification primers (Table S5). The resulting Tn10 construct is referred to as deletometer in the following text.

The deletometer pool. A pool of strains carrying the deletometer at various chromosomal locations was constructed in a strain with a *moaA*. The pNK974 plasmid carrying the gene expressing the Tn10 transposase protein constitutively was inserted into this strain using P22 transduction. A purified transductant was selected at random and verified to harbour the pNK974 plasmid. Cells were grown o/n at 37ºC in LB-broth supplemented with 100mg/L ampicillin. From the overnight culture, cells were diluted 1:100 and grown to OD_{600}~0.5 before transduced with P22 lysate grown on the original deletometer strain carrying the deletometer on the F'128. The pool was generated from ~10,000 individual colonies that were resuspended in LB-broth containing 1mM EGTA and washed 4 times in LB-broth supplemented with 1mM EGTA. The pooled cells were resuspended in 1ml LB-broth supplemented with 100µl DMSO and frozen at -80ºC. From this mixture 10µl was used to start a new overnight culture from which a P22 phage lysate was made. Cells with *moaA* deleted from the

chromosome were transduced with this lysate and 50 colonies were chosen at random and purified from phage on EBU-agar plates.

Localization of random deletometer insertions. Twenty strains obtained from a transposon pool harboring the deletometer were picked at random and the localization of the deletometer was determined by semi-random PCR as described previously [5]. In short, the semi-random PCR consist of two steps of PCR. In the first step transposon specific and semi-random primers consisting of one specific part and one random part were used. In the second round of PCR another transposon specific and a primer consisting of the specific part of the semi-random primer are used. The PCR products were sequenced to determine the location of the mini-Tn101617 (tetR).

Verification of genes involved in chlorate resistance. Previous studies identified molybdenum as a cofactor for nitrate reductase [6], an enzyme that can convert chlorate to chlorite, which is a compound toxic for bacterial cells. As a result, any mutation inactivating nitrate reductase renders the cells chlorate resistant [6]. A set of seven insertions in the *S. typhimurium* chromosome that rendered cells chlorate resistance were isolated and named *chlA-G* [1,7]. Two of these genes, *chlA* and *chlD*, were mapped to 18 centisomes of the *E. coli* chromosome and found to take part in molybdenum biosynthesis and uptake [1]. The *chlA* gene has more recently been identified as the *moa*-operon, located approximately 40 and 20kb from the *mod*-operon in the *S. typhimurium* and *E. coli* chromosomes, respectively. The *moa*-operon is involved in the biosynthesis of molybdenum, the cofactor of nitrate reductase. The *chlD* gene was more recently found in a gene operon named *mod* that is involved in molybdenum uptake pathway [8]. Decreased accumulation of molybdenum has been seen in cells lacking any of the *mod*-genes [9] and inactivation of the *mod*-operon has been shown to make cells chlorate resistant in *E.coli* [10,11]. However, in initial experiments when we inactivated the whole *mod*-operon through linear transformation in *S. typhimurium*, this did not make the cells chlorate resistant. Instead, inactivation of the *moaA* gene alone resulted in chlorate resistance. These results have some precedence in the literature as a study regarding large chromosomal deletions in the *chl-gal* region of *S. typhimurium* also failed to find any deletions that were chlorate resistant and that only lacked the *mod*-operon, whereas all deletions lacking the *moa*-operon were found chlorate resistant [12]. In conclusion, even though inactivation of

the *mod*-operon makes cells chlorate resistant in *E. coli*, the same resistance requires inactivation of the *moaA* gene in *S. typhimurium*.

Calculation of energetic cost of running *E. coli* flagella. The number of protons used per flagellum per revolution equals: Number of stators per motor x number of steps per revolution x number of protons per stator per step = (12) x (25) x 2 = 600 protons per flagellum per revolution. The number of stators per motor is based on data from [13], number of steps per revolution from [14,15] and the number protons per stator per step from [16,17]. The motors turn at about 300 revs per second at 37°C [18,19], so each motor will use about 300 x 600 = 180,000 protons per second and in a cell with five flagella the motors together would use about 900,000 protons per second. It has been estimated that aerobically respiring cells pump out about 20,000,000 protons per second [20]. Thus, from these values we would estimate that the flagella use roughly 900,000 / 20,000,000, or 4.5%, of the proton "budget" of the cell. Because protons drive flagella this calculation is in terms of proton usage, but in terms of an opportunity cost (i.e. ATP that might otherwise have been made using these protons), the conclusion is the same; i.e., around 4.5% of the energy budget is going to turn the flagella instead of making ATP.

Take-over time for an adaptive deletion mutation. Consider a bacterial population of constant effective size N. Deletion mutations in a particular genomic region occur with rate k_{del} per genome replication. The selection coefficient for such a deletion is s ($s > 0$ assumed). Here we consider all possible deletions that are sufficiently large to have a positive effect on growth rate and assume that their sizes are constrained in a similar manner as the ones we have measured (Table S1). Thus, the deletion mutations considered are of a certain class from a certain genomic region, but they are not all identical in their genotype. Estimates for k_{del}, can therefore be taken from the measured values from the different regions. What is the mean time before the deletion mutation has taken over 50% of the population?

The calculations below follow very closely those presented in the supplementary material of Gullberg et al. (2011). Deletion mutants will arise in the population with rate $k_{del}N$. With probability $s/[1\text{-}\exp(\text{-}sN)] \approx s$, a new deletion mutant will survive and spread in the population. Thus, the mean time for the first surviving mutant to appear is $1/(k_{del}Ns)$ if none were present initially. Thereafter its presence in

the population will grow deterministically. The fraction of mutants at time t will change according to

$$\frac{df}{dt} = k_{del}(1-f) + sf(1-f) \qquad \text{(S1)}$$

As back mutations for a deletion are very unlikely, they are not included in this formalism. Given a starting fraction $f_0 = 1/N$ in the population, the solution to this equation is

$$f(t) = \frac{f_0 + (k_{del}/s)\left[1-(1-f_0)\exp\left(-(s+k_{del})t\right)\right]}{f_0 + (k_{del}/s) + (1-f_0)\exp\left(-(s+k_{del})t\right)} \qquad \text{(S2)}$$

The time $t = T_{50}$ when $f(t) = 0.5$ is given by

$$T_{50} = \frac{1}{s+k_{del}}\ln\left(\frac{(1+2k_{del}/s)(1-f_0)}{f_0 + k_{del}/s}\right) \approx \frac{1}{s}\ln\left(\frac{sN}{s+k_{del}N}\right) \qquad \text{(S3)}$$

The approximation holds in the reasonable limit where $k_{del} << s$.

Thus, the total time is the sum of the stochastic waiting time for the first surviving mutant to appear and the deterministic growth from $f_0 = 1/N$ to $f = 1/2$ thereafter.

$$T_{50}^{0} = \frac{1}{k_{del}Ns} + \frac{1}{s}\ln\left(\frac{sN}{k_{del}N+s}\right) \qquad \text{(S4)}$$

We have tested these results using a more complete stochastic model, a Moran model with overlapping generations. Individuals carrying the mutation have a faster growth rate by a factor $1+s$. The probability, $P_n(t)$, that the population contains n mutations at time t obeys the master equations

$$\frac{dP_n}{dt} = k_{n-1}^{+}P_{n-1} - \left(k_n^{+} + k_n^{-}\right)P_n + k_{n+1}^{-}P_{n+1}; \quad 0 \le n \le N \qquad \text{(S5)}$$

with $P_{-1} = P_{N+1} = 0$. Adapting the model by Berg and Kurland (2002) to the present situation, the rates of change can be expressed as

$$\begin{aligned} k_n^{+} &= \left[(1+s)n + k_{del}(N-n)\right]\frac{N-n}{N+1} \\ k_n^{-} &= \frac{(1-k_{del})(N-n)n}{N+1} \text{ for } n < N/2 \qquad \text{(S6)} \\ k_n^{-} &= 0 \text{ for } n = N/2 \end{aligned}$$

As we want to calculate the time to reach $n = N/2$, it is assumed that this step is irreversible such that $k_n^{-} = 0$ for $n = N/2$. The rate k_n^{+} is accounts for all the ways that

the number of mutants in the population could change from n to n+1 as follows: with rate 1+s one of the n mutated cells divides, or with rate 1 one of the N-n wt cells divides and picks up the mutation with probability k_{del}. This leaves the population with N+1 cells out of which with n+1 are mutated. To keep the population at a constant size N, one cell is picked at random to die; with probability (N-n)/(N+1), this is a wt cell. Similarly for k_n^- : with rate 1 one of the N-n wt cells divides and with probability 1-k_{del} does not pick up the mutation; then with probability n/(N+1) a mutated cell is chosen to die.

The first passage time can be calculated from the sum of the residence times in the states below $n = N/2$

$$T_{50} = \sum_{n=0}^{N/2-1} \tau_n \qquad \text{(S7)}$$

The residence time in each state is determined by $\tau_n = \int_0^\infty P_n(t)dt$. Assuming that the system starts in the state $n = 0$, and for simplicity of notation setting $m = N/2$ -1, one finds by integration of the master equations (Eq. S5) the following recursive relations for τ_n:

$$\begin{aligned} \tau_m &= \frac{1}{k_m^+}, \\ \tau_{m-j} &= \frac{1}{k_{m-j}^+}\left(1 + k_{m+1-j}^- \tau_{m+1-j}\right) \text{ for } j = 1,...m \end{aligned} \qquad \text{(S8)}$$

For the parameter ranges considered here, (s = 0.005, 0.05; N = 10^8, 10^9; $10^{-10} \leq k_{del} \leq 10^{-8}$), the take-over time is mostly dominated by the second term in Eq. (S4) and is not so much dependent on k_{del} or N. For $k_{del}N < 0.1$ or so, the stochastic waiting time (the first term in Eq. (S4)) starts to dominate. Thus, as long as $k_{del}N > 0.1$ or so, the actual value of the deletion rate matters very little to the take-over time (Figure S4). As a comparison we can calculate the mean time for a neutral deletion to penetrate half the population. This can be done from the stochastic model above after setting s = 0. We can also give a more intuitive estimate. It is well known that the probability that a neutral mutation takes over a population of size N is just 1/N; similarly, the probability that it penetrates half the population is 2/N. It appears in the population with rate $k_{del}N$, and consequently the stochastic waiting time for the first surviving (i.e. one that actually reaches at least 50% of the population) neutral deletion is 1/2k_{del}. This is the dominant contribution and a comparison with the result from the

full stochastic model shows that this estimate holds very well. In the parameter ranges considered here, this is on the order of $10^7 – 10^{10}$ generations.

Normalization of deletion rates. The experiments measure the rate of all deletions that include at least ca. 2 kbp that carry the inserted reporter genes plus (at most) the parts of the surrounding chromosome that do not carry any vital sequences. In this way the measured rate has two components: an intrinsic rate constant related to the propensity in each region to form a deletion and the number of all possible deletions that could form which satisfy these minimum and maximum requirements in range. The effective deletion rate for some gene(s) on the chromosome will have similar contributions and depend on an intrinsic rate in the region as well as on the required and possible ranges that would contribute to positive selection. In the discussion here it can be assumed that both the intrinsic rates and the allowed ranges are similar to the ones that were measured. Thus, the estimated effective deletion rate for some gene(s) may be on the order of $k_{del} \sim 10^{-8} – 10^{-9}$. Allowing for a ten-fold larger or smaller k_{del}, we get the results in Figure S4. It should be noted, that except for very small deletion rates, the take-over time of a deletion is largely independent of k_{del} and in fact, for the calculation of fixation times here, it matters little what the allowed ranges of the deletions are.

Assuming that the maximum range on each side of the reporter genes is L1 and L2 bp, respectively, the total number of allowed deletions in this region will be L1 x L2. The minimum values of L1 and L2 will be the observed maximum ranges, M1 and M2, in the sample of sequenced deletions. On the assumption that all allowed deletions are equally likely to have occurred, it can be shown [21] that the Minimum Variance Unbiased Estimates for L1 and L2 based on the observed maximum ranges M1 and M2 are

$$\mathrm{L1}_{est} = \frac{n+1}{n}\mathrm{M1} - 1, \quad \mathrm{L2}_{est} = \frac{n+1}{n}\mathrm{M2} - 1 \qquad \text{(S9)}$$

where n is the number of sampled deletions in the region considered. Here we have assumed that the reporter genes occupy 2 kbp in the central region of the inserted transposon. Thus there are at least 4.9 kbp on each side where deletion points are allowed. To this we have added the maximum range of the deleted chromosome on each side to form M1 and M2. The intrinsic deletion rate in each region can be estimated by dividing the observed rate with the estimated number of acceptable

deletions, $L1_{est}$ x $L2_{est}$. This gives an average intrinsic rate in the 11 regions considered of $k_D = 2.2 \times 10^{-11}$ per $(\text{kbp})^2$ per generation. If this intrinsic rate were operating continually everywhere over the genome of length, ~5×10^3 kbp, it would lead to a total of 3×10^{-4} deletions per generation. Most of these deletions would be lethal and this rate would therefore also correspond to the death rate due to deletions. This is probably a gross overestimate as very long deletions may be unlikely to form. Also, the average k_D has a large contribution from one high rate that is very uncertain and also may be operating only in a small region of the genome.

It should be stressed that the estimates from Eq. (S9) are very uncertain, particularly for small n. In fact, with 95% confidence it can be inferred that $M1 \leq L1 \leq M1 \times 20^{1/n}$ (i.e. a one-sided confidence interval). Thus for n=1, there is a significant possibility that the true values of both L1 and L2 could be up to 10-fold larger than their best estimates from Eq. (S9). To get the corresponding confidence limit for L1 x L2 we have simulated the process of picking n random deletions with allowed ranges L1 and L2 to the left and right and counted the fraction of times that the ratio $R = L1 \times L2/L1_{est} \times L2_{est}$ is larger than a certain number. In 5% of the cases, the ratio R is found to be larger than R_{95} = 29, 4.8, 2.8, 2.1, 1.8, and 1.62 for n = 1, 2, 3, 4, 5, and 6, respectively. The estimated intrinsic deletion rates at each insertion position together with the 95% confidence limit are plotted in Figure S1.

Supplementary References

1. Miller JB, Amy NK (1983) Molybdenum cofactor in chlorate-resistant and nitrate reductase-deficient insertion mutants of Escherichia coli. J Bacteriol 155: 793-801.
2. Koskiniemi S, Andersson DI (2009) Translesion DNA polymerases are required for spontaneous deletion formation in Salmonella typhimurium. Proc Natl Acad Sci U S A 106: 10248-10253.
3. Nilsson AI, Koskiniemi S, Eriksson S, Kugelberg E, Hinton JC, et al. (2005) Bacterial genome size reduction by experimental evolution. Proc Natl Acad Sci U S A 102: 12112-12116.
4. Datsenko KA, Wanner BL (2000) One-step inactivation of chromosomal genes in Escherichia coli K-12 using PCR products. Proc Natl Acad Sci U S A 97: 6640-6645.

5. Pranting M, Negrea A, Rhen M, Andersson DI (2008) Mechanism and fitness costs of PR-39 resistance in Salmonella enterica serovar Typhimurium LT2. Antimicrob Agents Chemother 52: 2734-2741.
6. Pateman JA, Cove DJ, Rever BM, Roberts DB (1964) A Common Co-Factor for Nitrate Reductase and Xanthine Dehydrogenase Which Also Regulates the Synthesis of Nitrate Reductase. Nature 201: 58-60.
7. Stewart V, MacGregor CH (1982) Nitrate reductase in Escherichia coli K-12: involvement of chlC, chlE, and chlG loci. J Bacteriol 151: 788-799.
8. Hemschemeier S, Grund M, Keuntje B, Eichenlaub R (1991) Isolation of Escherichia coli mutants defective in uptake of molybdate. J Bacteriol 173: 6499-6506.
9. Scott D, Amy NK (1989) Molybdenum accumulation in chlD mutants of Escherichia coli. J Bacteriol 171: 1284-1287.
10. Adhya S, Cleary P, Campbell A (1968) A deletion analysis of prophage lambda and adjacent genetic regions. Proc Natl Acad Sci U S A 61: 956-962.
11. Maupin-Furlow JA, Rosentel JK, Lee JH, Deppenmeier U, Gunsalus RP, et al. (1995) Genetic analysis of the modABCD (molybdate transport) operon of Escherichia coli. J Bacteriol 177: 4851-4856.
12. Alper MD, Ames BN (1975) Positive selection of mutants with deletions of the gal-chl region of the Salmonella chromosome as a screening procedure for mutagens that cause deletions. J Bacteriol 121: 259-266.
13. Reid SW, Leake MC, Chandler JH, Lo CJ, Armitage JP, et al. (2006) The maximum number of torque-generating units in the flagellar motor of Escherichia coli is at least 11. Proc Natl Acad Sci U S A 103: 8066-8071.
14. Sowa Y, Rowe AD, Leake MC, Yakushi T, Homma M, et al. (2005) Direct observation of steps in rotation of the bacterial flagellar motor. Nature 437: 916-919.
15. Nakamura S, Kami-ike N, Yokota JP, Minamino T, Namba K (2010) Evidence for symmetry in the elementary process of bidirectional torque generation by the bacterial flagellar motor. Proc Natl Acad Sci U S A 107: 17616-17620.
16. Braun TF, Blair DF (2001) Targeted disulfide cross-linking of the MotB protein of Escherichia coli: evidence for two H(+) channels in the stator Complex. Biochemistry 40: 13051-13059.
17. Sato K, Homma M (2000) Functional reconstitution of the Na(+)-driven polar flagellar motor component of Vibrio alginolyticus. J Biol Chem 275: 5718-5722.
18. Meister M, Lowe G, Berg HC (1987) The proton flux through the bacterial flagellar motor. Cell 49: 643-650.
19. Magariyama Y, Sugiyama S, Muramoto K, Maekawa Y, Kawagishi I, et al. (1994) Very fast flagellar rotation. Nature 371: 752.
20. Harold FM, Maloney PC (1996) Energy transduction by ion currents. in Escherichia coli and Salmonella typhimurium: cellular and molecular biology eds Neidhardt F C, Ingraham J L, Low K B, Magasanik B, Schaechter M, Umbarger H D (ASM Press, Washington DC) pp 283–306.
21. Johnson RW (1994) Estimating the size of a population. Teaching Statistics 16: 50-52.
